# Supplementary material for: Public perceptions and support for introduced microbes to combat hospital-acquired infections and antimicrobial resistance
Source: PLoS One. 2025 Oct 8;20(10):e0332578. doi: 10.1371/journal.pone.0332578 (PMC12507289; doi:10.1371/journal.pone.0332578)
Supplement: S1 File — https://example.com/file1-supplementary-materials. (DOCX) [file pone.0332578.s001.docx]

Public Perceptions and Support for Introduced Microbes to Combat Hospital-Acquired Infections and Antimicrobial Resistance

### Supplementary Materials

#### Description of Microbiome Engineering of the Built Environment

The full description of microbiome engineering of the built environment that was presented to respondents at the beginning of the survey is provided below.

“In recent years, a new area of study has emerged at the intersection of microbiology, architecture, and environmental science – microbiome engineering in the built environment. The “built environment” includes indoor spaces that humans have created, like houses and office buildings.

A “microbiome” is a community of small life forms interacting with each other in a particular place. It is a tiny ecosystem made up of living things, including microbes. This ecosystem can exist in various environments, like on or within animals and plants, on surfaces in built environments, or in water and soil. Microbes, including bacteria, viruses, and other small creatures, can have both beneficial and harmful effects— some contribute to vital bodily functions, while others can lead to illnesses.

This new field of study is exploring how microbiomes could be intentionally controlled, designed, and introduced into human-made structures like homes, office buildings, or hospitals to achieve specific benefits like improving air quality or public health.

However, as with any new science and technology issue, the introduction of microbiomes may have unintended negative consequences, which are being evaluated as well.”

#### Description of Introduced Microbes into Hospital Sinks as a Potential Application of ME

Participants were asked to read the following description of a potential use of microbiome engineering and respond to questions specific to this application.

“Hospitals face a difficult challenge to stop harmful microbes that can lead to infections. Typical treatments are not always effective because some microbes have become resistant, meaning they don't respond to medicines or treatments. As a solution, there is growing interest in the idea to purposefully introduce microbiomes in hospital sinks to support the growth of good microbes and control the growth of harmful ones.”

#### Independent Variables

The full list of items used to create the independent variables are provided below. The response scale was 1 (strongly disagree), 2 (disagree), 3 (neither disagree nor agree), 4 (agree), and 5 (strongly agree) for all of the items below, with the exception of the emotion questions, which were measured on a scale of 1 (not at all), 2 (low), 3 (moderate), 4 (high), and 5 (very high).

**Prior information-seeking about microbiome engineering**

1. I have actively sought information or education about microbiome engineering in the context of indoor environments.

**Familiarity with microbiome engineering**

1. I am familiar with the concept of microbiome engineering.
2. I am aware of the different techniques and technologies used in microbiome engineering.
3. I feel well-informed about how microbiome engineering can impact air quality and health in indoor spaces.

**Perceived knowledge of microbiome engineering**

1. I have a basic understanding of how microbiome engineering can be applied.
2. I feel confident in my knowledge of the potential benefits of microbiome engineering in enhancing the built environment.
3. I believe I have a good grasp of the potential risks associated with microbiome engineering.
4. I feel knowledgeable about the current research and developments in microbiome engineering for improving indoor spaces.

**Desire to learn more about microbiome engineering**

1. I would like to learn more about microbiome engineering and its applications.

**Negative emotions about microbiome engineering**

While reading the previous information, to what degree did you experience…

1. Disgust
2. Distaste
3. Anger
4. Fear
5. Anxiety
6. Helplessness
7. Nervousness

**Positive emotions about microbiome engineering**

While reading the previous information, to what degree did you experience…

1. Excitement
2. Satisfaction
3. Relief
4. Enthusiasm
5. Optimism
6. Wonder
7. Contentment

**Threat appraisal of antimicrobial resistance and hospital-acquired infections**

1. Existing microbial resistance in hospitals poses a severe health risk. (severity perception)
2. I would be at risk of infection from harmful microbes if I were in a hospital. (susceptibility perception)

**Coping appraisal of introduced microbes**

1. I am confident that using introduced microbiomes will contribute to a safer and more hygienic healthcare environment. (response efficacy perception of IM)
2. I trust that healthcare facilities can effectively manage and maintain the safety of introduced microbiome solutions in sinks.
3. I trust that healthcare regulatory agencies will provide clear guidelines for the safe use of introduced microbiomes in hospital sinks.

**Secondary risk perception of IM**

1. I am concerned about the potential risks and safety issues associated with the use of microbiomes in hospital sinks.
2. I have concerns that the introduction of microbiomes in sinks may have unintended consequences on water quality or health.

#### Dependent Variables

The full list of items used to create the dependent variables are provided below. The response scale was 1 (strongly disagree), 2 (disagree), 3 (neither disagree nor agree), 4 (agree), and 5 (strongly agree).

**Model 1: Support for IM in hospital sinks to reduce AMR and HAIs**

1. I am open to the idea of using introduced microbiomes if they are shown to be effective in reducing microbial resistance.
2. I would support the implementation of introduced microbiome solutions in hospital sinks if they were part of a comprehensive strategy to combat microbial resistance.

**Model 2: Support for rigorous evaluation and testing of IM**

1. I think that the introduction of microbiomes in hospital sinks should be subject to rigorous testing and evaluation.

**Model 3: Support for healthcare staff and patient education of IM**

1. I believe that healthcare staff and patients should be educated about the use and benefits of introduced microbiomes in sinks.
