# Supplementary material for: Public perceptions and support for introduced microbes to combat hospital-acquired infections and antimicrobial resistance
Source: PLoS One. 2025 Oct 8;20(10):e0332578. doi: 10.1371/journal.pone.0332578 (PMC12507289; doi:10.1371/journal.pone.0332578)
Supplement: S2 File — (PDF) [file pone.0332578.s002.pdf]

NC STATE UNIVERSITY

Sponsored Programs and Regulatory Compliance
Institutional Review Board (IRB)
Jennie Ofstein, Director
IRB-Director@ncsu.edu

Campus Box 7514
2701 Sullivan Drive
Raleigh, NC 27695
P: 919.515.8754

From: Jennifer Ofstein, IRB Director
North Carolina State University
Institutional Review Board

Date: November 29, 2023
Title: Microbiome Technologies in Focus: A Public Opinion Survey
IRB#: 26517

Dear Dr. Cummings,

The research proposal named above has received administrative review and has been approved on 11/28/2023 as exempt from the policy as outlined in the Code of Federal Regulations (d.2). Provided that the only participation of the subjects is as described in the proposal narrative, this project is exempt from further review. This approval does not expire, but any changes must be approved by the IRB prior to implementation.

NOTE:

1. This committee complies with requirements found in Title 45 part 46 of The Code of Federal Regulations. For NC State University projects, the Assurance Number is: FWA00003429.

2. Any changes to the research must be submitted and approved by the IRB prior to implementation.

3. If any unanticipated problems occur, they must be reported to the IRB office within 5 business days.

4. When all research activities (including analysis of identifiable data) are completed, please submit a study closure request.

5. As a part of routine best practices, the NC State IRB office engages in post approval monitoring activities. please refer to the NC State University IRB website.

Please forward a copy of this letter to your faculty sponsor, if applicable.

Thank you.

Sincerely,

Jennifer Ofstein

Jennifer B. Ofstein, Ph.D., IRB Director
North Carolina State University
Institutional Review Board (IRB)
irb-director@ncsu.edu
919.515.8754
