# Supplementary material for: Public perceptions and support for introduced microbes to combat hospital-acquired infections and antimicrobial resistance
Source: PLoS One. 2025 Oct 8;20(10):e0332578. doi: 10.1371/journal.pone.0332578 (PMC12507289; doi:10.1371/journal.pone.0332578)
Supplement: Attachment — Submitted filename: Reviewer_Response_Table Hospital sink paper 31JUL25.docx [file pone.0332578.s003.docx]

# Response to Reviewers and Editorial Comments

We thank the editors and reviewers for their insightful comments—we have addressed them all in the revised manuscript and provide granular responses to all comments below. Your feedback has greatly improved the quality and clarity of the work, for which, we are thankful. Best regards,

CC

| Editor/Reviewer Comment | Author Response |
| --- | --- |
| EDITOR |  |
| Please ensure that your manuscript meets PLOS ONE's style requirements, including those for file naming. | Thank you, will do |
| Please ensure that you include a title page within your main document. Could you therefore please include the title page into the beginning of your manuscript file itself, listing all authors and affiliations. | We have added this. |
| Please provide additional details regarding participant consent. In the ethics statement in the Methods and online submission information, please ensure that you have specified (1) whether consent was informed and (2) what type you obtained (for instance, written or verbal, and if verbal, how it was documented and witnessed). If your study included minors, state whether you obtained consent from parents or guardians. If the need for consent was waived by the ethics committee, please include this information. | All procedures involving human participants were approved by the North Carolina State University Institutional Review Board (IRB #26517). Informed consent was obtained from all participants before participation. Participants were informed of the voluntary nature of the study and provided digital consent before beginning the survey. The IRB approved a waiver of documentation of written consent due to minimal risk and the online administration of the survey. No minors were included. We have updated the text accordingly. |
| We note that the grant information you provided in the ‘Funding Information’ and ‘Financial Disclosure’ sections do not match. When you resubmit, please ensure that you provide the correct grant numbers for the awards you received for your study in the ‘Funding Information’ section. | The Center for Precision Microbiome Engineering (PreMiEr) is a National Science Foundation (NSF)-funded Engineering Research Center (ERC) (Award # 2133504) |
| Thank you for stating the following financial disclosure:  “National Science Foundation”  Please state what role the funders took in the study. If the funders had no role, please state: "The funders had no role in study design, data collection and analysis, decision to publish, or preparation of the manuscript."  If this statement is not correct you must amend it as needed.  Please include this amended Role of Funder statement in your cover letter; we will change the online submission form on your behalf. | Thank you, we added this text to the disclaimer, “The funders had no role in study design, data collection and analysis, decision to publish, or preparation of the manuscript." |
| We note that you have indicated that there are restrictions to data sharing for this study. For studies involving human research participant data or other sensitive data, we encourage authors to share de-identified or anonymized data. However, when data cannot be publicly shared for ethical reasons, we allow authors to make their data sets available upon request. For information on unacceptable data access restrictions, please see http://journals.plos.org/plosone/s/data-availability#loc-unacceptable-data-access-restrictions.  Before we proceed with your manuscript, please address the following prompts:  a) If there are ethical or legal restrictions on sharing a de-identified data set, please explain them in detail (e.g., data contain potentially identifying or sensitive patient information, data are owned by a third-party organization, etc.) and who has imposed them (e.g., a Research Ethics Committee or Institutional Review Board, etc.). Please also provide contact information for a data access committee, ethics committee, or other institutional body to which data requests may be sent. | The data underlying this study cannot be shared publicly due to ethical and legal restrictions imposed by the North Carolina State University Institutional Review Board (IRB). Although the dataset is de-identified, it contains potentially sensitive information related to participants’ health beliefs, religious practices, political orientations, and emotional responses to biotechnological interventions—data that, in combination, could pose a risk of deductive disclosure. The IRB determined that public release of the dataset, even in de-identified form, could not ensure adequate protection of participant privacy given the nature of the topics studied and the granularity of some variables. This determination aligns with established ethical standards in human subjects research that prioritize respect for persons, confidentiality, and the principle of minimizing harm. Access to the de-identified data may be requested on a case-by-case basis for qualified researchers who agree to strict confidentiality protocols and data use agreements. Requests should be directed to:  North Carolina State University Institutional Review Board  Email: irb-director@ncsu.edu  Phone: +1 (919) 515-4514  Website: https://research.ncsu.edu/compliance/irb/ |
| Please include your full ethics statement in the ‘Methods’ section of your manuscript file. In your statement, please include the full name of the IRB or ethics committee who approved or waived your study, as well as whether or not you obtained informed written or verbal consent. If consent was waived for your study, please include this information in your statement as well. | We have updated the text to read as follows, “This study was reviewed and approved by the North Carolina State University Institutional Review Board (IRB Protocol #26517). All participants provided informed consent prior to participation. Because the study posed minimal risk and was conducted online, the IRB approved a waiver of written documentation of consent. Participants provided digital consent after reading a disclosure statement describing the study’s purpose, procedures, and their rights as research participants. No personally identifiable information was collected, and all responses were stored and analyzed in de-identified form.” |
| We note that Figure 1 in your submission contain copyrighted images. All PLOS content is published under the Creative Commons Attribution License (CC BY 4.0), which means that the manuscript, images, and Supporting Information files will be freely available online, and any third party is permitted to access, download, copy, distribute, and use these materials in any way, even commercially, with proper attribution. For more information, see our copyright guidelines: http://journals.plos.org/plosone/s/licenses-and-copyright. | We removed the copyrighted Microsoft Word icons and replaced them with Canva Free Content License icons that we edited in order to add our own creativity. You can read the Free Content License agreement here: <https://www.canva.com/policies/content-license-agreement/>. We ensured each of the three icons was covered by the Free Content License. Please see sections 2 and 6 for relevant information about the Free Content License. |
|  |  |
| Reviewer 1 |  |
| The work by Cummings and collogues encapsulates an interesting investigation of the factors influencing one’s attitudes to using microbes as a preventative in preventing hospital acquired infections. There are many errors in the introduction where papers are cited that do not accurately support their claims. This reduces the reviewer’s enthusiasm for the work and their trust in the author’s presented results. | We have updated a few of the references to better support the claims made in this paper. |
| - Personally, I would reduce the number of abbrevations. It makes the text overly verbose. Too many to keep track of. | While we understand the concern, we have chosen to retain key abbreviations to preserve clarity and consistency with terminology common to microbiome engineering, infection control, and public health research. We have, however, reviewed the manuscript to ensure that all abbreviations are defined upon first use and that any non-essential abbreviations have been removed to minimize potential confusion. |
| - Reference to Rodrigues et al 2025 does not support using engineered bacteria, at no point in the cited study were probiotics used, however it was noted that women who were hospitalized were at a higher risk of acquiring an infection. | We have updates this to Wright’s 2025 work: Wright, K. (2025, March 13). The Future of Microbiome‑Based Therapeutics. European Medical Journal, 10[1]:35-39, https://doi.org/10.33590/emj/CTGY2726 |
| - Reference to Fady once again does not support the claim being made "For example, bioengineered waste lignocellulosic biochars have been shown to sequester MDR bacteria, reducing hospital contamination and minimizing antibiotic- resistant outbreaks (Fady et al., 2024).” | We have updated the claim to appropriately reflect the Fady et al work. It now reads, “precision-engineered waste lignocellulosic biochars have been shown to sequester multidrug‑resistant clinical isolates in model wastewater systems (removing up to 94% of Pseudomonas aeruginosa and 85% of Staphylococcus aureus) — though their efficacy in clinical or hospital environments has not yet been evaluated” |
| - This paper is not about phages at all. Discusses nanoparticles to treat infections but does not mention either of the points it is meant to support “Additionally, phage therapy and microbiome restoration techniques have gained traction as alternative interventions, showing promise in targeting antimicrobial-resistant bacterial strains without relying on traditional antibiotics (Ahmed et al., 2025).” Phage therapy has existed as a concept since the early 1900s, there should be at least a few references which would be better used to support this claim. | We have deleted this reference and adjusted the text. |
| - Another instance of an improper citation: “Recurrent bacteremia in the setting of Pseudomonas endocarditis of the tricuspid valve and indwelling medical devices”, cited work is case report, regarding biofilm formation on a medical device. No review of supplying probiotics to outcompete pathogens as suggested by the quoted line. | Thank you, we have corrected the text and added another citation that better supports the claims as follows, “Although recurrent infections associated with Pseudomonas and biofilm formation on medical devices remain a serious clinical challenge (Miller et al., 2024), emerging studies suggest that introduced non-pathogenic microbes may help outcompete pathogenic strains in certain contexts. For example, experimental trials using engineered bacterial consortia have demonstrated success in displacing Klebsiella pneumoniae and other multidrug-resistant pathogens in controlled environments (Sheth et al., 2022).”  Sheth, R. U., Cabral, V., Chen, S. P., & Wang, H. H. (2022). Manipulating bacterial communities by in situ microbiome engineering. Nature Reviews Microbiology, 20(5), 365–378. https://doi.org/10.1038/s41579-021-00641-1 |
| - There is no Cummings 2025 in the works cited. I assume that this is in reference to the manuscript itself, which seems odd if not misleading: “So far there have been limited studies of U.S. public attitudes on microbiome engineering in the built environment. In the first survey of built microbiome engineering and public attitudes, attitudes toward microbes and microbiome engineering were discovered to be shaped by a complex interplay of scientific literacy, emotional responses, media influence, and demographic factors (Cummings, Landreville, & Kuzma, 2024; Cummings, Landreville, & Kuzma, 2025)”. | Thank you for pointing this out—it is a reference to our team’s recent work and we have added the proper citation to the reference list as follows,  Cummings CL, Landreville KD and Kuzma J  (2025) Natural vs. genetically engineered  microbiomes: understanding public attitudes  for indoor applications and pathways for  future engagement.  Front. Genet. 16:1560601.  doi: 10.3389/fgene.2025.1560601 |
| - It would be interesting to see if one’s prior scientific knowledge was in anyway predictive of their thoughts on the microbiome in therapy, perhaps including this information in the model would provide interesting insights as to whether education is actually helpful in increasing the public’s perception of microbiome based therapeutics | While this is a compelling area for research, it falls outside the scope of the current study. Interestingly, prior research has consistently shown that increasing scientific knowledge alone—sometimes referred to as the “scientiation” approach—does not reliably lead to more positive attitudes toward emerging biotechnologies. Studies in science communication and public understanding of science have demonstrated that emotional responses, trust in institutions, and cultural values often play a more significant role in shaping public perceptions than factual knowledge alone (e.g., Cummings et al., 2024; Kahan, 2015; Allum et al., 2008). Nonetheless, we agree that understanding how specific types of knowledge may interact with other attitudinal drivers would be a valuable direction for future research. |
|  |  |
| Reviewer 2 |  |
| Cummings and colleagues present a very thorough, well written, and interesting assessment of Americans’ attitudes towards introducing engineered microbiomes into hospital sinks to combat antimicrobial resistance and hospital associated infections. The study is timely as AMR and HAI are major issues facing the US healthcare system and innovative strategies are needed to combat them. Additionally, deploying engineered microbiomes in the built environment is in early stages, making now an ideal time for assessing public attitudes so that they may inform future implementation strategies and promote success. I think the manuscript can be improved by adding some detail/clarifying information, tightening up some of the language to describe survey respondent characteristics, and including a discussion of the study’s limitations. My specific suggestions are below. | Thank you for the positive feedback! |
| Major comments  1. I think it would be nice to give a little detail/context on why the study/survey focused on introduced microbiomes specifically deployed in sinks (as opposed to other parts of the hospital built environment). | We fully agree and have added clarifying language in the manuscript to explain the focus on hospital sinks as the deployment site for introduced microbiomes. It know reads as follows, “This study focuses specifically on hospital sinks as the target site for introduced microbiomes due to their well-documented role as persistent reservoirs for multidrug-resistant organisms such as *Pseudomonas aeruginosa, Klebsiella pneumoniae*, and *Acinetobacter baumannii*. These pathogens frequently colonize sink drains and plumbing systems, where they can form biofilms, exchange resistance genes, and contribute to repeated contamination events within clinical environments. Sinks represent one of the few built-environment surfaces in hospitals currently being explored for targeted microbial interventions, including probiotic and synthetic consortia-based approaches. As such, they offer a realistic and policy-relevant use case for gauging public support for microbiome engineering in infection control.” |
| 2. I think the last sentence of the introduction – very clearly stating the overall research question – is really great and sets the reader up nicely for the rest of the manuscript! (no revisions requested here) | Thanks! |
| 3. Are there any notable examples/success stories of deploying engineered/introduced microbiomes in other built environment settings (healthcare or otherwise)? If yes, I would consider giving a brief overview of these in the literature review section, along with any lessons learned that may be relevant to the current study. Alternatively, are there any notable failures with relevant lessons learned? | We appreciate the reviewer’s thoughtful suggestion to include examples of successful or unsuccessful deployments of engineered or introduced microbiomes in built environments. While this is an important area of future inquiry, the field remains nascent, with few published cases of real-world implementation in healthcare or other operational settings. Most existing studies are limited to laboratory trials, pilot-scale interventions, or theoretical models. Given this limited empirical base, and in keeping with the scope of the current study, we have not added additional examples to the manuscript.  However, we emphasize that this work is anticipatory in nature and aligns with the principles of Responsible Research and Innovation (RRI) by proactively assessing public perceptions during the early stages of technology development. Rather than retrospectively evaluating public reactions to mature deployments, our study aims to inform the design, governance, and communication strategies of microbiome-based interventions before they reach widespread clinical use. In this way, we hope to contribute to more socially responsive innovation pathways as the field evolves. |
| 4. The language used to describe sex/gender, political orientation/party identification, and religiosity/church attendance is not internally consistent throughout the manuscript, and the alternative terms that are used have meaningful differences. Here are some specific clarifying questions and suggestions I have:  a. I really like that you give a full description of all participants’ reported gender in the methods! From those data, it seems like the survey asked about gender and not sex? If yes, terminology used later in the manuscript to refer to ‘females’ and ‘biological sex’ should be revised to refer to women and gender. If the survey assessed gender and sex, that should be stated in the methods, and the manuscript should clearly identify which variable is being discussed and use congruent terminology (e.g., ‘female’ to describe sex and ‘woman’ to describe gender).  b. The methods section defines the bounds of the party identification scale as ‘strong Republican coded’ and ‘strong Democrat coded’. Later, the results refer to ‘conservative political orientation’. In the current (and recent) American sociopolitical context, ‘Republican’ and ‘conservative’ are not interchangeable, and I think whichever language is intended to be used with the party identification scale should be used throughout the manuscript.  c. Did the church attendance scale explicitly assess ‘church’ attendance, or did it ask about attending any variety of religious/worship service or any variety of house of worship? If it only asked about church, I don’t think ‘religiosity’ or ‘religious attendance’ should be used to describe this independent variable, and it should instead be referred to as church attendance. | a. On the survey, participants were asked to select all the biological sex and gender categories that applied to them. The categories were: male, female, non-binary/genderqueer, transgender, prefer not to say, and other. All of those categories are reported in the Method section. We corrected any confusion surrounding the use of gender vs. sex throughout the manuscript.  b. The survey used the terms Republican and Democrat on the 7-point scale rather than conservative and liberal. We corrected all references to conservative to Republican. We also changed the name of the variable from “political orientation” to “political party identification” to more accurately describe our variable.  c. The survey asked specifically about church attendance so the terms religiosity and religious attendance were removed and changed to church attendance. |
| 5. In the methods or results, can you state how age and gender were operationalized in the models? Specifically, was age continuous or binned? How were individuals who did not share their gender and individuals identifying as nonbinary, genderqueer, trans, and ‘no label’ included in models? | Age was a continuous variable. Since we have a mix of biological sex and gender variables that were included in the survey as “select all that apply,” we decided to pick one for the regressions, and we chose male. Males were coded as 1 and all participants who did not chose male were coded as 0. We made this clear in the revised manuscript. |
| 6. Did you examine relationships among the dependent variables? I’m curious if any of the dependent variables are significant predictors of each other | In the current study, our analytic approach treated each outcome—support for implementation, evaluation, and education—as conceptually distinct but related dimensions of public engagement. As suggested by the reviewer, we examined the correlations among the three DVs. They are all significantly positively correlated with one another. Additionally, three regressions were run, with each DV being predicted by the other two. In each of the three regressions, the two remaining DVs (serving as the IVs now) significantly positively predicted the respective DV. |
| 7. Given the partial overlap of significant predictors between the three models, have you considered including a venn diagram of which ones are/aren’t shared across models, perhaps as a panel b for Figure 1? I think this could be a nice visualization, but also it may get clunky with more text than a typical venn diagram. | We explored this idea during the writing of the results, and, as you surmised, it indeed got “clunky” fairly quickly. We landed on Fig 1 as the most parsimonious summary visualization of the results and we hope you still find it of value. |
| 8. Can you include a limitations paragraph in the discussion? A couple limitations that I think deserve some discussion are: the survey doesn’t speak to hospital workers’ attitudes (out of the scope of this study but nonetheless important); the survey population has a higher proportion of respondents over 65 and of white respondents than the census estimate; and people’s personal histories related to hospital stays (e.g., recent/frequent/long hospital stays, death of a loved one in hospital) may meaningfully impact their attitudes, but these data were not collected (alternatively, if these data were collected, I think it would be really interesting to include them in the current analysis!). | We appreciate this feedback and fully agree, the new limitations paragraph reads as follows, “This study has several limitations that should be noted. First, while the sample was nationally representative on several key demographic indicators, it included a slightly higher proportion of respondents over age 65 and individuals identifying as white compared to recent U.S. Census estimates. These demographic differences may influence generalizability, particularly in relation to age- and race-related variation in trust, health experience, or familiarity with emerging biotechnologies. Second, the survey did not include healthcare workers, whose frontline experiences and institutional perspectives are likely to shape attitudes toward microbiome interventions in distinct ways. Future work should examine how perceptions vary between the general public and clinical stakeholders. Third, we did not collect data on participants’ personal hospital histories (e.g., recent or frequent admissions, experiences with HAIs, or the hospitalization of loved ones), which may significantly shape their emotional responses or perceived vulnerability. Incorporating these experiential variables in future research could enhance our understanding of how direct exposure to healthcare environments influences support for microbiome-based innovations.” |
| Minor comments  1. Can you add line numbers in the revised draft? | Done! |
| 2. Can you include interquartile ranges in the text alongside standard deviations? | We chose not to make this addition as it is not standard practice for reporting. |
| 3. There are a few spots where I’d like a bit of clarification:  a. When the methods refer to a ‘composite item’ of multiple questions, does this just mean that response values were averaged across those questions, and the average was used as a predictor?  b. You define the bounds of the 6-point scale used to assess church attendance (1 – rarely attends; 6 – attends weekly). For other scales, it may be easy to assume what the rest of the scale values are defined as, but I’m not sure it’s as intuitive for church attendance. Can you define all six scale values in the methods?  c. Can you state the p value cutoff for determining significance in the methods or results?  d. In the results, does ‘strongest predictor’ refer to the predictor with the largest-magnitude beta estimate or the smallest p value?  e. In the methods or Table 1 footnotes, can you state what test was used to determine significance of changes in R2 across successive blocks of predictors?  f. In the methods, can you state what software programs, packages, functions, and versions were used in the analyses? | a. Yes, a composite score (or composite item or composite variable) is just an average of the respective survey questions. The composite variable was then used as the predictor, where indicated in the paper.  b. All response options for the church attendance question have been added to the manuscript.  c. Significance was determined at the p < .05 level. We also added the following note to Table 1: * *p* < .05, ** *p* < .01, *** *p* < .001.  d. The smallest p value was used, but if the p values were both < .001, then the beta estimate was used.  e. The F-change test was used and that detail is now added to the note under Table 1.  f. SPSS v. 29 was used and that detail was added to the Analytical Approach in the Method section. |
| 4. I’m not sure ‘change’ is the best word to use in Table 1 to describe increases in R2 across successive blocks of predictors. My understanding is that R2 should increase (or stay the same) with each successive block of predictors (i.e., monotonic increases in R2). ‘Change’ implies that R2 may decline with added blocks of predictors, and only presenting the change in R2 for blocks 2-5 may be a bit confusing. I’d suggest either using a different word than ‘change’, or presenting the overall R2 for each block alongside the change from the prior block. | While we agree that this is a bit of strange terminology, it is the most commonly used phrasing for reporting the increase in R² across successive blocks of predictors in Table 1. While we agree that the term “change” may suggest the possibility of a decrease—despite the monotonic nature of R² in hierarchical regression models—it remains a widely used and recognized term in social science and behavioral research for describing incremental increases in explained variance (ΔR²). |
| 5. The bottom of Figure 1 appears to be cut off. | Thanks-- it seems clean and complete in our copy—we can check with the Editors should the paper be fully accepted for publication. |
| 6. In the first paragraph of the discussion, can you either add a citation for the last part following sentence (starting at ‘potentially’), or state that it is a hypothesis: ‘Interestingly, prior information-seeking and familiarity with ME were often negatively associated with support, suggesting that surface-level or critical exposure may lead to skepticism, potentially due to the framing of microbiome interventions or conflation with genetic modification and synthetic biology.’ | We have revised the statement to now read as follows, “Interestingly, prior information-seeking and familiarity with ME were often negatively associated with support. One possible explanation is that surface-level or critically framed exposure, such as through media emphasizing risks, may contribute to public skepticism, perhaps due to the conflation of microbiome engineering with genetic modification or synthetic biology. While this remains a hypothesis, it highlights the need for further research into how the framing and context of information shape public attitudes toward emerging biotechnologies.” |
|  |  |
| Reviewer 3 |  |
| Well-written manuscript highlighting the importance of microbiome engineering to combat HAI. Authors investigated (with an RRI) approach, how people perceive the use of microbes in these "sterile" hospital environments to promote the growth of beneficial microbes. | Thank you! |
| 1. If the participants wanted to know the exact science behind the introduction of beneficial microbes to hospital sinks, were they provided an explanation? | We appreciate the reviewer’s question. All participants received the same standardized information about the proposed use of introduced microbiomes in hospital sinks, which was designed to provide a general, accessible description of the intervention. No additional or individualized scientific explanation was offered beyond this brief description. This approach was intentional to ensure consistency across respondents and to reflect the type of information the general public might realistically encounter in early-stage public communication efforts. It also helped to minimize the risk of researcher-induced bias by avoiding differential framing or elaboration that could unintentionally influence participant responses. |
| 2. Were the surveys completed in person (at a hospital, clinic, at home, etc)? | The surveys were administered online through YouGov’s national panel. Participants completed the survey remotely using their personal devices, typically at home or another location of their choosing. No surveys were conducted in person or at clinical or hospital sites. This mode of administration is consistent with standard practices for large-scale, nationally representative public opinion research. |
| 3. Is it possible to gain more insight into societal value and ethical considerations of microbiome engineering/HAI by surveying individuals in predominately Black, AA and/or Latinx communities? | Absolutely! In the United States (and elsewhere) these populations have historically faced disparities in healthcare access, exposure to environmental health risks, and trust in biomedical interventions—all of which may shape perceptions of emerging technologies like microbiome engineering. While our current nationally representative sample includes respondents from diverse racial and ethnic backgrounds, it was not specifically designed or powered to analyze within-group perspectives or culturally specific ethical concerns. Future research using targeted sampling approaches or community-engaged methods would be well-suited to examine how these communities assess the risks, benefits, and governance of microbiome-based interventions, particularly in relation to trust, equity, and structural health inequalities. We have added this to the limitations section, “Future studies should consider targeted sampling of underrepresented populations, including Black, African American, and Latinx communities, to explore how culturally specific experiences and historical health disparities shape perceptions of microbiome engineering and hospital-based interventions.” |
| 4. Historically, how influential has the public perception been on developing novel techniques in hospitals? | Very interesting question! Historically, public perception has played a complex but increasingly influential role in the development and adoption of novel techniques in hospitals and broader healthcare systems. While early biomedical innovations were often introduced with limited public input, recent decades have seen growing recognition of the importance of public trust, ethical acceptability, and sociocultural alignment in shaping the success or failure of new medical technologies. High-profile cases—such as vaccine hesitancy to novel mRNA vaccines, demonstrate that public perception can significantly influence funding priorities, regulatory scrutiny, and clinical uptake.  In hospital settings, technologies perceived as invasive, poorly explained, or insufficiently tested will likely encounter resistance from both patients and healthcare providers, especially when trust in institutions is fragile. Conversely, we believe that innovations that are introduced transparently, aligned with patient values, and supported by meaningful public engagement are more likely to be accepted and integrated into clinical workflows. These dynamics underscore the relevance of anticipatory research, such as our study, which aims to assess public attitudes early in the development process to inform more ethically robust and socially responsive innovation pathways. |
